# Left Ventricular Apical Long-Axis Length and Ejection Fraction: A Proof-of-Concept Analysis

**DOI:** 10.7759/cureus.97972

**Published:** 2025-11-27

**Authors:** Khalid Sawalha, Aakash Rana, Lana Alamat, Munes Albadaineh, Landon Bruich, Srikanth Vallurupalli, Angel Lopez Candales

**Affiliations:** 1 Cardiovascular Disease, University of Arkansas for Medical Sciences, Little Rock, USA; 2 Cardiometabolic Medicine, University of Missouri-Kansas City School of Medicine, Kansas City, USA; 3 Internal Medicine, Central Arkansas Veterans Healthcare System, Little Rock, USA; 4 Internal Medicine, Jordan Hospital, Amman, JOR; 5 Internal Medicine, University of Arkansas for Medical Sciences, Little Rock, USA

**Keywords:** echocardiography, left ventricular apical length, left ventricular ejection fraction, left ventricular systolic function, modified simpson’s method

## Abstract

Background: Accurate estimation of left ventricular ejection fraction (LVEF) is vital in cardiology practice for diagnostic, prognostic, and management decisions. The biplane Simpson method is the standard but can be subject to limitations, particularly in cases of poor image quality or when subjective interpretation is involved. This study aims to evaluate whether LVEF can be reliably estimated using simpler apical four-chamber views without the need for complex calculations.

Methods: A retrospective review of 260 echocardiographic studies from the University of Arkansas for Medical Sciences database was conducted. LVEF was calculated using the Simpson’s biplane method and compared with longitudinal apical LV length measurements in systole and diastole. Statistical analyses, including regression and receiver operating characteristic (ROC) curves, were used to determine the relationship between LVEF and apical LV lengths.

Results: Patients were divided into two groups based on LVEF values (>50% normal, <50% reduced). Longitudinal apical LV length measurements demonstrated significant differences between the groups. Delta changes in LV length (>14 cm) showed high sensitivity (94.2%) and specificity (95.7%) in identifying normal LVEF. ROC analysis revealed that end-systolic lengths ≤7.3 cm could predict normal LVEF with good accuracy.

Conclusions: Simple measurements of longitudinal apical LV length changes between systole and diastole can serve as a reliable surrogate for LVEF, offering a rapid bedside alternative to the biplane Simpson method. These findings highlight the potential for streamlining LVEF estimation, particularly in settings where time and resources are limited.

## Introduction

Accurate estimation of left ventricular ejection fraction (LVEF) is the cornerstone of day-to-day cardiology and echocardiography practice for diagnostic and prognostic implications of cardiovascular disease and its clinical management [1-11].

Estimation of LVEF, expressed as a fraction of the ejected LV chamber volume in systole (stroke volume) in relation to the volume of blood in the LV at the end of diastole, using the biplane Simpson, is unfortunately subject to inter-observer variability, which likely increases with declining image quality [12-15]. Despite the availability of different imaging modalities that can be used for the determination of LVEF, echocardiography remains the most widely used because it is rapid, portable, radiation-free, and highly cost-effective, allowing routine and repeated LVEF assessment at the bedside with sufficient diagnostic accuracy for clinical decision-making [15-19]. Other modalities include cardiac MRI, which is the reference standard for volumetric EF; cardiac CT with retrospective gating; radionuclide techniques such as multigated acquisition (MUGA); gated single-photon emission computerized tomography (SPECT) myocardial perfusion imaging; and invasive contrast left ventriculography performed during coronary angiography. 

Regardless of which modality is used, each has its limitations that can potentially affect accurate LVEF calculation. Echocardiography is no exception; particularly, poor endocardial border resolution, foreshortening, and the presence of wall motion abnormalities or distortion of the LV cavity can result in inaccurate estimation [15]. Furthermore, subjective interpretations can affect LVEF estimations. Consequently, the American Society of Echocardiography has provided practice guidelines that encourage the use of objective measurements that can standardize and provide accurate as well as reproducible LVEF assessments, such as the biplane Simpson’s method of disks, end-diastolic and end-systolic volume quantification, and when image quality is suboptimal, contrast-enhanced echocardiography, as well as 3D echocardiographic volumetric analysis, which reduces geometric assumptions and inter-observer variability [15]. These methods shift LVEF assessment from visual estimation to quantifiable, reproducible parameters [15].

Unfortunately, many still rely on subjective assessments that become even more problematic in cases when transthoracic acoustic interrogation is hindered by obesity, chronic obstructive pulmonary disease, pectus excavatum, the presence of draining chest tubes, bandages, breast implants, or tissue expanders, and simply limited space between ribs [15-20]. In addition, the widespread use of handheld ultrasound units, which often lack the ability to estimate biplane LVEF, has led to increasing subjective estimation.

Thus, the need for prompt and simple bedside LVEF determinations without the need for additional offline calculations exists. While small and hyperdynamic left ventricles can be easily differentiated from dilated chambers with markedly reduced LV contractility, difficulty in differentiating cases in which LVEF is mildly reduced or simply low normal is common. The latter becomes extremely relevant based on recent observations from Kondo et al. that patients’ clinical characteristics appear to diverge at a threshold of around 40% to 50% [21].

To that end, our study intends to answer two specific questions: determine if LVEF determinations can be provided only using four-chamber end-systolic and end-diastolic apical measurements when compared to the well-validated Simpson’s biplane method; determine if LV apical length measurements can be used as surrogate measures of LVEF without the need for tracing the endocardial cavity for disk summation.

## Materials and methods

A retrospective review of our echocardiographic database at the University of Arkansas for Medical Sciences was conducted to identify complete studies that, in addition to including all objective measurements routinely collected in our laboratory, also required good image quality without apical foreshortening.

Studies were excluded if patients were not in sinus rhythm, ectopy was present, thick papillary muscles, presence of pulmonary hypertension with distortion of the LV cavity, absence of adequate orthogonal views, presence of greater than mild pericardial effusion, or if studies were limited and performed by cardiology trainees.

This retrospective study received approval from the Institutional Review Board at the University of Arkansas for Medical Sciences and did not require a signed consent form.

Two-dimensional transthoracic echocardiography (TTE) studies were performed using commercially available systems (Vivid 9, GE Medical Systems, Milwaukee, WI). Images were obtained in the left lateral decubitus position with the patient in the supine position using a 3.5-MHz transducer. Standard two-dimensional, color, pulsed, and continuous-wave Doppler data were digitally acquired in gently held end-expiration and saved in regular cine loop format for subsequent offline analysis (EchoPAC version 111.0.00 (GE Vingmed Ultrasound AS, Horten, Norway)). Statistical analysis for the data was performed using Microsoft Excel (Microsoft Corporation, Redmond, Washington, United States).

**Figure 1:**
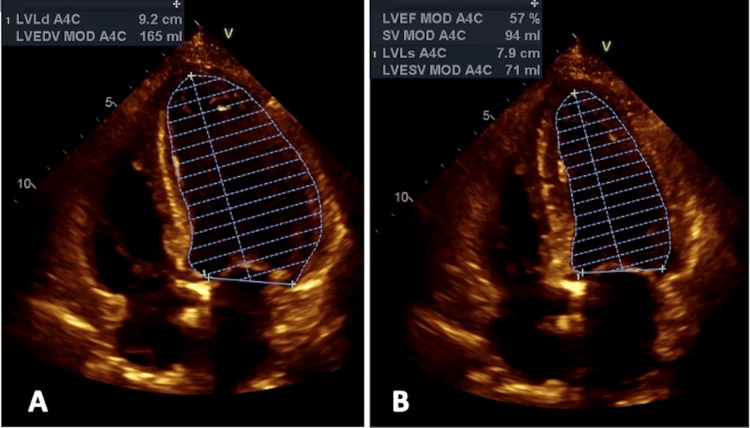
Apical longitudinal four-chamber views at end-diastole (A) and end-systole (B), with the LV measurements obtained LV: left ventricular

The following echocardiographic and Doppler measures were obtained in all patients included in the final analysis. Apical LV four-chamber longitudinal length measurements were obtained from non-foreshortened views, as previously described [15,22,23]. Specifically, both apical longitudinal end-systolic and end-diastolic LV measurements were obtained from the LV apex to the plane described by the mitral annulus, as shown in Figure 1.

In addition, the following two calculations were also made: \[ \Delta \text{ change} = \text{LV end-diastolic apical length} - \text{LV end-systolic apical length} \] \[\text{Fractional change} =\frac{\left(\text{LV end-diastolic apical length} - \text{LV end-systolic apical length}\right) \times 100\%}{\text{LV end-systolic apical length}}\]

In terms of the LV, EF was calculated using the modified Simpson's biplane method, as recommended by the American Society of Echocardiography [15]. Additional measures of LV systolic function also used in this study included mitral annular plane systolic excursion (MAPSE) obtained using M-mode and peak systolic (s’) velocity using tissue Doppler imaging (MA TDI s’) of the lateral mitral annulus, as previously described [15,24,25]. LV outflow tract velocity time integral (LVOT VTI) measurements were also obtained by placing the pulsed Doppler sample volume in the LV outflow tract below the aortic valve and recording the velocity measured in cm/s, which correlates well with cardiac output [15,26].

Left atrial (LA) volumes were calculated using the biplane area-length formula, while LV mass calculations were determined using M-mode echocardiography, as previously described [15]. Both measurements were corrected for body surface area (BSA) and expressed as left atrial volume index (LAVI) and left ventricular mass index (LVMI).

The maximal tricuspid regurgitation systolic velocity value was measured from multiple windows using continuous wave Doppler, and the highest velocity was used to estimate pulmonary artery systolic pressures using the modified Bernoulli equation [27]. An estimate of the mean right atrial pressure was then used to make the final calculation of pulmonary artery systolic pressures by determining the diameter and collapse index of the inferior vena cava with inspiration [27].

Measurements for each studied variable are shown as mean + standard deviation with their respective range of values obtained in our studied population. Unpaired data results were compared using the two-tailed Student’s t-test. A stepwise multiple linear regression analysis was then used to determine which echocardiographic variables best discriminate LVEF to determine the specific relationship that exists between LVEF and longitudinal apical LV lengths. Receiver operating characteristic (ROC) curves and areas under the curves were then examined and compared by the method of Hanley and McNeil to determine which cut-off values were most discriminative of normal LVEF for longitudinal apical LV lengths [28]. Finally, interobserver variabilities were measured between the modified Simpson’s biplane, standard computer measurements, and our measured longitudinal apical LV lengths obtained from four-chamber end-systolic and end-diastolic views. These readings were performed without knowledge of each other’s readings. Analysis of the differences between these values was calculated using an inter-rater agreement statistic (Kappa) to evaluate the agreement between two readings with a 95% confidence interval technique. P-values of <0.05 were statistically significant.

## Results

For this proof-of-concept analysis, data from 260 echocardiographic studies were analyzed, and measurements with range values for all pre-specified data points are presented in Table 1.

**Table 1:** Echocardiographic data for the entire studied population LAVI: left atrial volume index; LVMI: left ventricular mass index; LVESV: left ventricular end-systolic volume; LVEDV: left ventricular end-diastolic volume; LVEF: left ventricular ejection fraction; MAPSE: mitral annular plane systolic excursion; MA TDIs: mitral annular tissue Doppler imaging systolic velocity; LVOT VTI: left ventricular outflow tract velocity time integral; LV: left ventricle; PASP: pulmonary artery systolic pressure

Variable	Mean	Range
LAVI (ml/m^2^)	24 ± 10	7-65
LVMI (g/m^2^)	91 ± 38	32-255
LVESV (ml)	18 ± 11	5-84
LVEDV (ml)	36 ± 10	12-93
LVEF (%)	53 ± 17	10-80
MAPSE (cm)	1.3 ± 0.4	0.4-2.4
MA TDI s’ (cm/s)	8.7 ± 3.2	2-23
LVOT VTI (cm)	20 ± 6	5-38
PASP (mmHg)	16 ± 13	5-68
LV end-systolic length (cm)	6.9 ± 1.3	3-12
LV end-diastolic length (cm)	8.5 ± 1.0	5-12

The mean age of these patients was 53 ± 15 years (range: 18 to 89 years old), with 134 males (52%) and a mean BSA of 1.93 ± 0.26 m^2^ (range: 1.1 to 2.9 m^2^).

To better assess the value of using longitudinal apical LV lengths to characterize LV systolic function, we divided the studied population into two groups based on the recent upper reference value of 50% for LVEF proposed by Kondo et al. [21]. Accordingly, Group 1 included 190 patients with normal LVEF, while Group 2 consisted of 70 patients with reduced LVEF. Patient and echocardiographic data for these two groups are shown in Table 2.

**Table 2:** Patient and echocardiographic variables of the two patient groups divided according to LVEF LAVI: left atrial volume index; LVMI: left ventricular mass index; LVESV: left ventricular end-systolic volume; LVEDV: left ventricular end-diastolic volume; LVEF: left ventricular ejection fraction; MAPSE: mitral annular plane systolic excursion; MA TDIs: mitral annular tissue Doppler imaging systolic velocity; LVOT VTI: left ventricular outflow tract velocity time integral; LV: left ventricle; PASP: pulmonary artery systolic pressure

Variable	Group 1 (n=190)	Group 2 (n=70)	P-value
Age (years)	51 ± 16	57 ± 13	0.005
BSA (m²)	1.93 ± 0.25	1.94 ± 0.27	0.8
LAVI (mL/m²)	21.1 ± 8.3	31.5 ± 11.3	0.00001
LVMI (g/m²)	77.4 ± 23.3	127.3 ± 42.9	0.00001
LVESV (mL)	12.5 ± 3.2	32.6 ± 12.2	0.00001
LVEDV (mL)	33.0 ± 6.3	43.9 ± 11.9	0.00001
LVEF (%)	62 ± 6	27 ± 11	0.00001
MAPSE (cm)	1.5 ± 0.3	0.8 ± 0.3	0.00001
MA TDI s’ (cm/s)	9.4 ± 2.8	5.7 ± 2.0	0.00001
LVOT VTI (cm)	21 ± 5	14.1 ± 5.1	0.00001
PASP (mmHg)	13 ± 10	25 ± 16	0.00001
LV systolic length (cm)	6.4 ± 0.9	8.3 ± 1.2	0.00001
LV diastolic length (cm)	8.3 ± 0.9	9.0 ± 1.1	0.00001

Group 1 patients were younger (p < 0.005) with a lesser proportion of male patients (46% vs. 67%; p < 0.001) and similar BSA values (p = 0.8) than Group 2 patients. As expected, Group 1 patients had statistically lower LV and LA dimensions with higher measures of LV systolic function than Group 2 patients.

**Figure 2:**
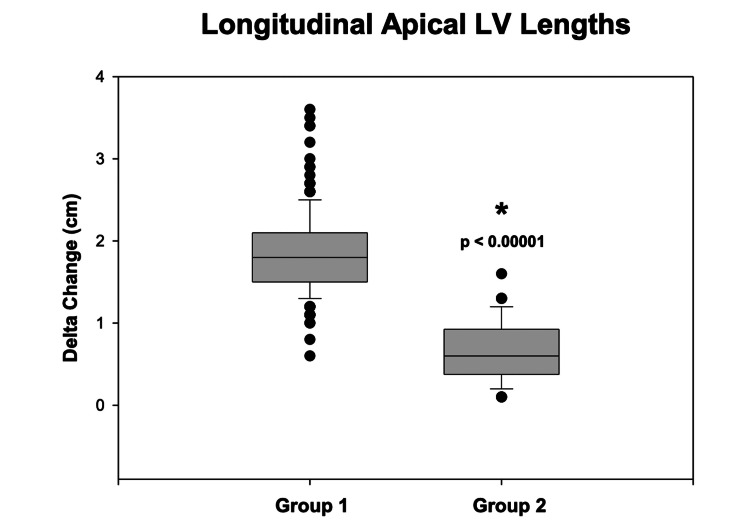
Delta change in longitudinal apical LV lengths between groups Box-and-whisker plot comparing the delta change in apical left ventricular (LV) lengths (in cm) between Group 1 (patients with normal LVEF) and Group 2 (patients with reduced LVEF). Group 1 shows significantly larger delta changes compared to Group 2 (p < 0.00001). LVEF: left ventricular ejection fraction

**Figure 3:**
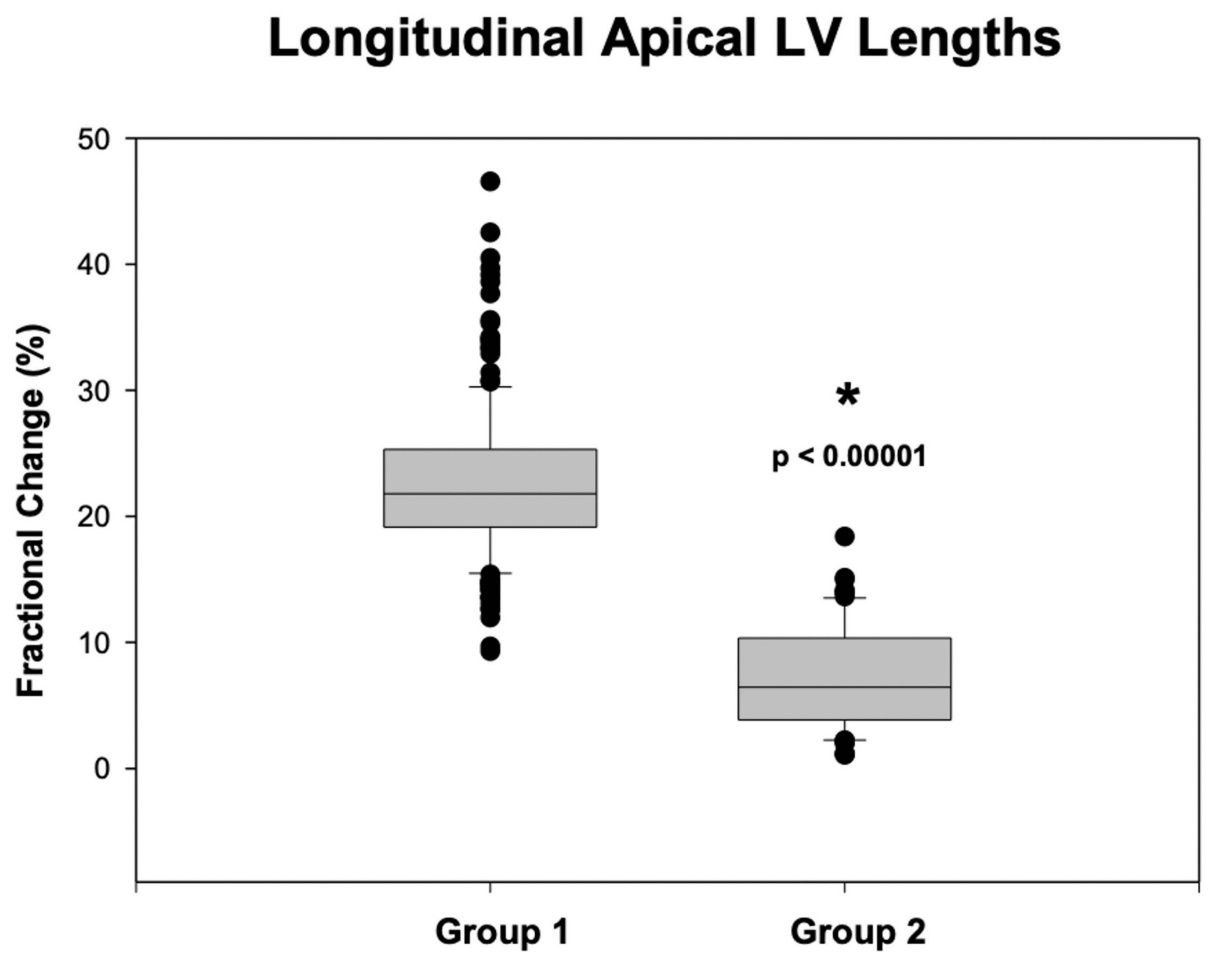
Fractional change in longitudinal apical LV lengths between groups Box-and-whisker plot showing the fractional change in apical LV lengths (in %) between Group 1 and Group 2. Group 1 exhibits a significantly higher fractional change than Group 2 (p < 0.00001), indicating more robust LV systolic function in patients with normal LVEF. LVEF: left ventricular ejection fraction

Longitudinal apical end-systolic (6.4 ± 0.9 cm; p < 0.00001) and end-diastolic (8.3 ± 0.9 cm; p < 0.00001) LV length measurements from Group 1 patients were significantly smaller compared to Group 2 (8.3 ± 1.2 cm and 9.0 ± 1.1 cm, respectively) patients. Based on these changes in end-systolic and end-diastolic longitudinal apical LV lengths, the calculated delta change resulted in significantly greater values for Group 1 patients (1.87 ± 0.50) compared to Group 2 (0.63 ± 0.37 cm; p < 0.00001, Figure 2). As seen in Figure 3, the fractional change in Group 1 was 23 ± 6% compared to 7 ± 4% in Group 2 (p < 0.00001).

Given these results, a multiple regression analysis was then performed to determine which of the measured echocardiographic variables best correlated with LVEF. LAVI, LVOT VTI, and delta change in longitudinal apical LV lengths were the best discriminators of LVEF (Table 3). Measurement of longitudinal apical LV end-systolic or end-diastolic lengths is comparable in their ability to discriminate LVEF when compared to MAPSE and MA TDI s’ velocity.

**Table 3:** Results of the multiple linear regression analysis of two-dimensional and spectral Doppler signals on the different measurements with regard to LVEF MAPSE: mitral annular plane systolic excursion; LAVI: left atrial volume index; LVMI: left ventricular mass index; MA TDIs: mitral annular tissue Doppler imaging systolic velocity; LVOT VTI: left ventricular outflow tract velocity time integral; LV: left ventricle. Multiple regression analysis was then performed to determine which of the measured echocardiographic variables best correlated with LVEF.

Independent variables	Coefficient	Std. error	P
MAPSE	5.5098	1.8893	0.0039
LAVI	-0.2563	0.05593	<0.0001
LVMI	-0.04124	0.01694	0.0156
MA TDI s	0.4709	0.2096	0.0255
LVOT VTI	0.5996	0.1074	<0.0001
LV systolic length	-12.4764	4.8458	0.0106
LV diastolic length	9.6598	4.0049	0.0166
Delta LV length	0.7910	0.08378	<0.0001

**Figure 4:**
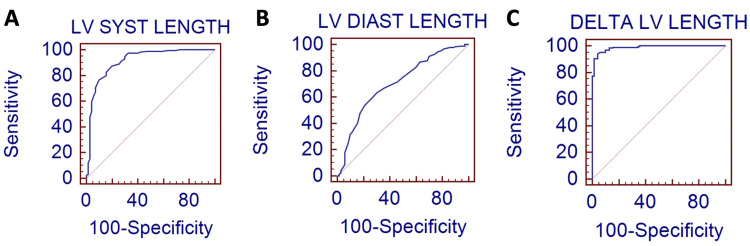
Receiver operating characteristic (ROC) curves for the predictive value of left ventricular (LV) measurements A: LV systolic length; B: LV diastolic length; C: delta change in LV length. The curves demonstrate the discriminative power of each measurement. The diagonal red line represents the line of no discrimination (sensitivity = specificity), and the area under the curve (AUC) provides a summary measure of the predictive performance for each metric. Delta LV length (C) shows the highest discriminatory ability with the steepest curve and AUC closest to one.

Receiver-operating characteristic curve analysis was then performed to identify the best value that would discriminate normal versus reduced LVEF in our studied population. These analyses were performed for both end-systolic and end-diastolic four-chamber view LV long axis length measurements, as well as for the delta change measure. In terms of the end-systolic LV length measure, a value of ≤ 7.3 cm identified patients with normal LVEF with a sensitivity of 87.4% and a specificity of 80% (AUC: 0.914, standard error 0.0226, 95% confidence interval 0.873 to 0.945 with a P < 0.0001) (Figure 4A). For end-diastolic LV length measures, a value of ≤ 8.5 cm also identified patients with normal LVEF; however, it had lower sensitivity (63.2%) and specificity (70%) with the following AUC: 0.705, standard error 0.0368, 95% confidence interval 0.645 to 0.760, and P < 0.0001, as shown in Figure 4B. Finally, the best measurement to discriminate LVEF was the delta change with a value of > 14 cm with a sensitivity of 94.2% and a specificity of 95.7% (AUC: 0.988, standard error 0.00496, 95% confidence interval 0.966 to 0.997 with a P < 0.0001) (Figure 4C).

Finally, interobserver variability analysis was conducted to determine the utility of end-systolic and diastolic LV chamber dimensions obtained from only four-chamber views to quantify LVEF. We compared measurements of these views to LVEF measurements obtained using the standard modified Simpson biplane method. A total of 50 consecutive samples were randomly selected, corresponding to an equal number of patients from both groups 1 and 2. This analysis showed a very good overall agreement (weighted kappa value of 0.825 with an SE of 0.028 with a 95% confidence interval of 0.771 to 0.880) between four-chamber end-systolic and end-diastolic LV chamber measurements to calculate LVEF compared to the standard LVEF assessments obtained using the modified Simpson biplane method.

## Discussion

While estimation of LVEF remains the gold standard for assessment of LV systolic function [1-11], the difficulty in the optimal methods of estimation and even the normal thresholds remains unclear [29].

Over the years, numerous large multicenter trials, including the Multicenter Postinfarction Research Group, the Coronary Artery Surgery Study Registry, the GISSI-3 trial (Gruppo Italiano per lo Studio della Sopravvivenza nell'Infarto Miocardico), and the Digitalis Intervention Group, reported the close relationship existing between declining LV systolic function and worse clinical outcomes [30-32]. Specifically, this relationship regarding the degree of LV systolic dysfunction is independent of the imaging modality used for its assessment [33].

As already stated, quick estimations of LVEF from small and hyperdynamic LVs are easily made from estimates made when dilated chambers are encountered with markedly reduced contractility. Unfortunately, differentiating mildly reduced from simply low normal LVEF can be challenging at times. Objective demarcation of this minimalistic difference has become extremely relevant based on recent data, distinguishing the important threshold of LVEF between 40% and 50% [21].

To that end, our study aimed to simplify LV systolic function estimation without the need for complex offline calculations by relying on useful methods that can provide quick and reliable bedside assessments. First, our results showed a very good overall agreement between four-chamber end-systolic and end-diastolic LV chamber measurements to calculate LVEF compared to the standard LVEF assessments obtained using the modified Simpson biplane method. Second, even though both longitudinal apical end-systolic and end-diastolic LV length measurements were significantly smaller in patients with normal LVEF, routine day-to-day measurements could be simplified by calculating the delta change between these two measures. We found that a value of > 1.4 cm had an extremely high sensitivity (94.2%) and specificity (95.7%) to identify patients with normal LVEF. 

Estimations of LVEF are largely affected by changes in preload, afterload, and heart rate, variables not routinely included in day-to-day clinical assessments. Current echocardiographic guidelines recommend using the modified Simpson biplane method to quantify LV volume and systolic function [15]. This biplane approach using two axes to globally assess LV function has retained its prominent role despite the inherent limitations, which include suboptimal endocardial definition, extensive wall motion abnormalities, geometric assumptions, adequate alignment of apical views without foreshortening, arrhythmias, and the influence of load-dependent factors on ventricular function assessment [34].

From a mechanistic perspective, a well-defined concept of the modified Simpson biplane method relies on the longitudinal apical LV length. Based on simple geometry, this longitudinal apical LV length corresponds to the major axis of an ellipse [35,36]. Furthermore, this major axis is responsible for defining its shape, size, and orientation, as well as attributing various unique properties to the ellipse [35,36]. The latter geometrical shape has been given to the LV and appropriately assumed as a prolate ellipsoid shape with a ratio of 1:2 minor-to-major axis [37]. Alterations in this shape suggest deleterious remodeling of the heart, with larger LV volumes predicting worse clinical outcomes [38]. This initial data advocated for the incorporation of sphericity measures into LV function assessments, which not only reflect the global underlying pathophysiology leading to LV dysfunction but also provide a pictorial representation of LV remodeling status [39]. Following the same geometric principle, when the deviation of the ellipse’s eccentricity approximates a circle, LV systolic dysfunction ensues, and the latter is in turn linked with adverse cardiovascular events [40,41]. Sphericity is an underutilized but validated measurement of LV geometry and remodeling. and longitudinal apical LV length is one of the measurements used in its calculation [42-44]. 

Therefore, these geometric principles suggest that a simple length measurement obtained from a non-foreshortened apical 4-chamber view, and its change between end-systolic and end-diastolic longitudinal LV lengths, can be considered a surrogate measure of LV systolic function and LVEF. This is particularly important in the era of handheld ultrasound, where simple linear measurements are feasible in most units. However, regional wall-motion abnormalities can distort true LV geometry and alter apical longitudinal shortening, meaning that apical length change becomes an unreliable surrogate for global LVEF in the presence of ischemic or segmental dysfunction; apical remodeling, aneurysm formation, or akinetic segments may preserve or exaggerate apparent apical length despite markedly reduced global systolic function, and echocardiographers should exercise caution when such abnormalities are present [42-44].

To our knowledge, this is the first study attempting to describe whether LV apical length measurements can be used as surrogate measures of LVEF without the need for tracing the endocardial cavity for disk summation. Even though these results would have been anticipated a priori, the weight of science relies on scientific validation. Despite the attributes of these two proven assumptions as useful measurements, we must acknowledge the following limitations. First, the sample size is small, a common limitation of proof-of-concept analyses. These results serve as the foundation for larger prospective studies that incorporate these easily obtained echocardiographic measurements and, ideally, contrast them to speckle tracing data, known to detect LV myocardial dysfunction despite apparently preserved LVEF estimates [45-47]. Second, the lack of prospective clinical follow-up limits our understanding of the prognostic implications of this measurement. Finally, the effect of valvular abnormalities as well as arrhythmias on these measurements is unknown.

## Conclusions

In conclusion, echocardiography plays a pivotal role in the day-to-day routine assessment of cardiac structure and function. We provide data suggesting that simple measurements of the change between end-systolic and end-diastolic longitudinal apical LV lengths can serve as a surrogate measure of LVEF. This geometric principle offers a rapid and accurate confirmatory measurement for the modified biplane Simpson’s LVEF calculation without requiring complex volumetric, tissue Doppler, speckle-tracking, or other offline analyses. Future studies should incorporate additional echocardiographic measures that reflect geometric changes in LV structure and function rather than relying solely on LVEF. The next steps include conducting a large prospective validation study, extending the assessment to patients with regional wall-motion abnormalities, and determining how these simple linear indices correlate with meaningful clinical outcomes.
